# Neuropathy-Associated HSPB1 Mutant Impairs Neuronal Mechanoadaptation and Axonal Regeneration

**DOI:** 10.3390/cells15131216

**Published:** 2026-07-03

**Authors:** Jiming Xie, Ronglin Han, Haidong Xu, Zhiyu Li, Jingyi Zhao, Ying Wan, Xianchao Pan, Juan Xing

**Affiliations:** 1 School of Basic Medical Science, Bengbu Medical University, Bengbu 233030, China; 20230199120034@stu.swmu.edu.cn (J.X.);; 2 School of Basic Medical Science, Southwest Medical University, Luzhou 646000, China; 3 Department of Pathology, West China Hospital, Sichuan University, Chengdu 610041, China; 4 Key Laboratory of Basic and Clinical Cardiovascular Diseases, Bengbu Medical University, Bengbu 233030, China

**Keywords:** Charcot-Marie-Tooth disease, HSPB1, mechanotransduction, substrate stiffness, axonal regeneration, transglutaminase

## Abstract

The small heat shock protein HSPB1 is a ubiquitously expressed mechanoresponsive chaperone essential for cytoskeletal remodeling under mechanical load. Mutations in HSPB1, including S135F, cause Charcot-Marie-Tooth (CMT) peripheral neuropathy, yet the mechanisms underlying the selective vulnerability of peripheral nerves remain enigmatic. Here we demonstrate that substrate stiffness is a critical determinant of HSPB1^S135F^-mediated neurodegeneration. Using stiffness-tunable polydimethylsiloxane (PDMS) substrates (1 kPa, 10 kPa, 2 MPa) and uniaxial cyclic stretch, we show that primary dorsal root ganglia (DRG) neurons and SH-SY5Y cells expressing HSPB1^S135F^ exhibit profound deficits in mechanoadaptation. On compliant substrates (10 kPa), HSPB1^S135F^ causes stretch-induced axon fragmentation and neuronal death, whereas HSPB1^WT^ confers robust neuroprotection. HSPB1^S135F^ also disrupts stiffness-directed neuritogenesis in differentiated SH-SY5Y cells: HSPB1^WT^-expressing cells show optimal axonal outgrowth and βIII-tubulin expression on 10 kPa substrates mimicking muscle tissue stiffness, while HSPB1^S135F^ mutants display disorganized focal adhesions and complete differentiation failure. Mechanistically, we uncover that HSPB1^S135F^ dysregulates stage-specific transglutaminase (TGase) expression—insufficient TGase during early neuritogenesis impairs filopodia stabilization, whereas aberrant TGase persistence at late stages constrains axon extension. Our findings establish HSPB1 as a biomechanical sensor that integrates ECM stiffness signals to coordinate peripheral nerve regeneration, and identify defective mechanoadaptation as a previously unrecognized pathomechanism in CMT. These results open new avenues for stiffness-targeted therapeutic strategies in peripheral neuropathy.

## 1. Introduction

Mechanical forces are fundamental regulators of cell behavior. From the beating heart to the stretching skin, cells throughout the body are continuously exposed to physical cues that must be perceived and transduced into biochemical signals to maintain tissue homeostasis [1,2]. The peripheral nervous system (PNS) is particularly mechanosensitive: peripheral axons are embedded in compliant extracellular matrices (ECM), experience repetitive stretch during limb movement, and must navigate stiffness gradients during development and regeneration [3,4]. Disruption of this mechanical dialogue is increasingly recognized as a contributor to neurodegeneration, yet the molecular machinery that confers mechanical resilience to peripheral neurons remains poorly understood.

The small heat shock protein HSPB1 (also known as mouse hsp25 and human hsp27) has recently emerged as a critical mechanoresponsive regulator [5]. In mouse fibroblasts subjected to uniaxial cyclic stretch, HSPB1 is phosphorylated by the p38 mitogen-activated protein kinase (MAPK) signaling cascade [6]. Phosphorylated HSPB1 translocates from a diffuse cytoplasmic pool to discrete actin filament structures, particularly the “comet tails” that emanate from focal adhesions—sites of elevated tension and dynamic actin polymerization [6,7]. This mechanically triggered recruitment is essential for stress fiber reinforcement, cell spreading, and the negative regulation of cell migration. Loss of HSPB1 in CRISPR/Cas9-edited fibroblasts abrogates the actin reinforcement response to stretch, whereas re-expression of wild-type (WT) HSPB1, but not a nonphosphorylatable mutant, fully rescues this deficit [7]. Thus, HSPB1 functions as a phosphorylation-dependent mechanotransducer that couples mechanical signals to cytoskeletal adaptation.

Beyond its role in cultured fibroblasts, HSPB1 is highly expressed in neurons and is upregulated after axonal injury, where it promotes neuronal survival and regeneration [8]. More than 30 missense mutations in HSPB1 are causatively linked to Charcot-Marie-Tooth (CMT) disease, the most common inherited peripheral neuropathy [9,10,11]. CMT-associated mutations, including the hotspot S135F substitution within the α-crystallin domain, cause a toxic gain-of-function characterized by aberrant binding to client proteins such as α-tubulin and SQSTM1/p62, leading to microtubule stabilization, autophagic flux impairment, and proteostatic collapse under stress [12,13,14]. Yet a central paradox remains: why do mutations in a ubiquitously expressed chaperone selectively devastate the PNS, while the central nervous system (CNS) is largely spared?

We hypothesized that the PNS-selective vulnerability of HSPB1 mutations reflects the unique mechanical demands placed upon peripheral neurons. Unlike CNS neurons, which are encased in the rigid skull and vertebral column, PNS axons traverse soft, deformable tissues and must continuously adapt to dynamic mechanical loads [15]. Emerging evidence indicates that peripheral nerves exhibit a high degree of mechanosensitivity, with neural cells sensing and responding to the mechanical properties of their microenvironment through a range of mechanosensitive molecules and mechanotransduction pathways that ultimately regulate cellular function and behavior [16,17]. Given the established role of HSPB1 in cellular mechanoadaptation, we reasoned that CMT-causing mutations might specifically impair the neuron’s ability to sense and respond to ECM stiffness and cyclic strain—a deficit that would be unmasked only in mechanically permissive microenvironments.

To test this hypothesis, we employed stiffness-tunable polydimethylsiloxane (PDMS) substrates that recapitulate the soft (1 kPa) stiffness of CNS tissue, the intermediate (10 kPa) stiffness of muscle, and the rigid (2 MPa) stiffness of traditional culture plastic [18,19]. Using primary dorsal root ganglia (DRG) neurons from HSPB1^WT^ and HSPB1^S135F^ transgenic mice, together with a complementary SH-SY5Y neuronal model system, we systematically interrogated three aspects of neuronal mechanobiology: (1) adaptive cytoskeletal remodeling under cyclic stretch, (2) stiffness-directed neurite outgrowth and focal adhesion assembly, and (3) stiffness-optimized neuronal differentiation. Here we report that the HSPB1^S135F^ mutation profoundly compromises each of these mechanoresponsive behaviors, causing axonal fragility, regenerative failure, and complete differentiation blockade specifically on physiologically relevant compliant substrates. Mechanistically, we identify stage-specific dysregulation of the retinoic acid (RA)-responsive enzyme transglutaminase (TGase) as a critical downstream effector linking the S135F mutation to aberrant cytoskeletal remodeling. Our findings redefine CMT pathogenesis as the product of a genetic lesion that disables a cell’s intrinsic mechanoadaptive capacity, and establish HSPB1 as a central node in the biomechanical circuitry that sustains peripheral nerve integrity.

## 2. Materials and Methods

### 2.1. Reagents and Antibodies

Polydimethylsiloxane (PDMS) membranes with defined stiffness were fabricated as previously described [19]. The following primary antibodies were used: anti-α-tubulin (Abcam, Cambridge, UK, ab18251), anti-vinculin (Sigma, St. Louis, MO, USA, V9131), anti-TGase (Abcam, EPR28142-86), anti-βIII-tubulin (Abcam, ab18207), anti-Flag (Sigma, F1804), anti-β-actin (Beyotime, Shanghai, China, AF0003), anti-Phospho-(Ser/Thr) Phe (Abcam, EPR26858-4). Rhodamine-phalloidin against F-actin was purchased from Thermo Fisher Scientific, Waltham, MA, USA, (R415). Alexa Fluor 488- and 594-conjugated secondary antibodies and DAPI were from Molecular Probes. Nerve growth factor (NGF), retinoic acid (RA), poly-D-lysine, and laminin were obtained from Sigma-Aldrich.

### 2.2. Transgenic Mice

Transgenic C57BL/6J mice expressing Flag-tagged human HSPB1^WT^ or HSPB1^S135F^ were generated by Shanghai Model Organisms Center (China), Shanghai, China. The nervous system specific Thy1.2 promoter was employed to restrict the expression of HSPB1^WT^/HSPB1^S135F^ in neurons [20]. All animal procedures were approved by the Institutional Animal Care and Use Committee of the Southwest Medical University (ID: 20180309044) and conducted in accordance with the Guidelines for the Care and Use of Laboratory Animals by the National Institutes of Health.

### 2.3. Primary DRG Neuron Isolation and Culture

Dorsal root ganglia (DRG) were dissected from 3-month-old transgenic mice, collected in ice-cold DMEM (high glucose), and digested with 0.25% trypsin-EDTA for 30 min at 37 °C with gentle agitation every 5 min. Digestion was terminated by addition of fetal bovine serum (FBS) to a final concentration of 10%. The cell suspension was centrifuged at 1500 rpm for 15 min, resuspended in DMEM containing 10% FBS and 1% penicillin-streptomycin, and filtered through a 70 μm cell strainer [21]. Neurons were plated onto PDMS membranes precoated with 100 μg/mL poly-D-lysine and 10 μg/mL laminin [22,23], and cultivated overnight in a humidified atmosphere of 5% CO_2_ at 37 °C. The following day, the medium was replaced with B-27™ Plus Neuronal Culture System (Gibco) supplemented with 1% glutamine, 1% penicillin-streptomycin, and 40 ng/mL nerve growth factor (NGF). Cultures were maintained for up to 1 week prior to fixation or stretch stimulation.

### 2.4. SH-SY5Y Cell Culture, Differentiation, and Stable Cell Lines

Neuroblastoma SH-SY5Y cells stably expressing Flag-tagged HSPB1^WT^ or HSPB1^S135F^ were generated by lentiviral transduction as previously described [14]. Cells were cultured in DMEM/high glucose supplemented with 15% FBS and 1% penicillin-streptomycin. For neuronal differentiation, RA-based protocol was adapted from Cheung et al. [24]. In this study, a three-stage protocol were employed to perform RA-induced differentiation of SH-SY5Y cells. Briefly, cells were pro-differentiated with 10 μM RA in DMEM containing 3% FBS for 48 h (stage 1), followed by 10 μM RA in DMEM containing 1.5% FBS for 72 h (stage 2). Subsequently, cells were cultured in neuronal medium (Neurobasal™ medium with 2% B-27, 1% GlutaMAX, 50 ng/mL NGF, and 10 μM RA) for up to 20 days (stage 3). Media were refreshed every 2–3 days.

### 2.5. Fabrication and Characterization of Stiffness-Tunable PDMS Substrates

PDMS membranes with defined elastic moduli were prepared by varying the base-to-crosslinker ratio and spin-coating conditions as described [19]. Briefly, for soft substrates, a 100:1 (*w*/*w*) ratio of PDMS base (Sylgard 184, Dow Corning, Midland, MI, USA) to crosslinker was used; for substrates mimicking muscle-like stiffness, an 80:1 ratio was applied; and for rigid substrates, a 10:1 ratio was used. The mixtures were degassed, spin-coated onto glass slides, and cured at 70 °C for 7 days. The Young’s modulus of resulting PDMS membranes were determined through compression tests of PDMS cylinders as previously described [19], yielding values of approximately 1 kPa, 10 kPa, and 2186 kPa (2 MPa), respectively. Prior to cell culture, the membranes were coated with 100 μg/mL poly-D-lysine and 10 μg/mL laminin for 4 h at 37 °C, and thoroughly washed with PBS.

### 2.6. Uniaxial Cyclic Stretch

A custom-built uniaxial stretch device was used as previously described [25,26]. For stretch experiments, PDMS membranes (18.5 cm × 0.5 cm) composed of a base layer (modulus of 2 MPa) and a top layer with defined stiffness were prepared. The top layer was obtained by spin-coating onto the base membrane using different base-to-crosslinker ratios (100:1, 80:1, and 10:1) as described above. The resulting bilayer membranes were clamped at both ends and subjected to cyclic elongation. Supraphysiological stretch of 5–20% in peripheral nerves resulting from trauma, aberrant limb positioning, or surgery is known to cause conduction blocks [27]. To model the effects of mechanical stress on neurons, DRG neurons were subjected to cyclic stretch of 10% strain at 0.1 Hz for 30 min, whereas SH-SY5Y cells were exposed to cyclic stretch of 5% strain at 0.2 Hz for 30 min. After stretch stimulation, the apparatus was stopped at the pre-stretch position, and membranes were either fixed immediately (Stretched group) or incubated for an additional 24 h in fresh medium at 37 °C (Recovered group). Unstretched controls were maintained on identical membranes under the same culture conditions. Each experiment was performed in at least three independent biological replicates.

### 2.7. Immunofluorescence Staining and Confocal Microscopy

Cells were washed gently with PBS, fixed with 4% paraformaldehyde in PBS for 40 min at 4 °C, and permeabilized with 0.25% Triton X-100 in PBS for 10 min at room temperature [28]. After blocking with 5% BSA in PBS for 1 h, cells were incubated with primary antibodies diluted in 1% BSA overnight at 4 °C, followed by incubation with Alexa Fluor-conjugated secondary antibodies and rhodamine-phalloidin for 1 h at room temperature. Nuclei were counterstained with DAPI. Coverslips or PDMS membranes were mounted with ProLong™ Gold Antifade reagent (Invitrogen, Carlsbad, CA, USA). Images were acquired using a Leica SP8 confocal microscope with a 63× oil-immersion objective (NA 1.40) and LAS X software. Maximum intensity projections of z-stacks are shown. Quantitative analysis of cell number, cell area, and aspect ratio was completed in ImageJ 1.46r (http://imagej.net).

### 2.8. Western Blot Analysis

Cells were lysed in RIPA buffer (50 mM Tris-HCl pH 7.4, 150 mM NaCl, 1% NP-40, 0.5% sodium deoxycholate, 0.1% SDS) supplemented with protease and phosphatase inhibitor cocktails (Roche). Protein concentrations were determined using the BCA assay (Pierce). Equal amounts of protein (25 μg) were resolved by 10–15% SDS-PAGE, transferred to nitrocellulose membranes, and blocked with 5% BSA in TBST for 1 h at room temperature. Membranes were incubated overnight at 4 °C with primary antibodies, washed, and incubated with IRDye 800CW– or 680RD–conjugated secondary antibodies (LI-COR) for 1 h [14]. Fluorescent signals were detected using the Odyssey CLx imaging system and quantified with Image Studio Lite software (LI-COR). β-actin served as a loading control.

### 2.9. Quantification of Neurite Outgrowth and Network Complexity

Neurite outgrowth was analyzed using the NeuronJ plugin for ImageJ [29]. For each condition, ≥3 randomly selected neurons from at least three independent experiments were traced. Neurite length per neuron and the number of primary neurites were recorded. Neuronal arborization complexity was assessed by Sholl analysis using the Sholl Analysis plugin for ImageJ [30]; concentric circles with 10 μm step increments were centered on the soma, and the number of intersections per radius was quantified.

### 2.10. Statistical Analysis

All quantitative data are presented as mean ± SD. For each independent experiment, technical replicates were averaged, and the resulting values (*n* = 3 biological replicates) were used for statistical comparisons. Statistical analyses were performed using GraphPad Prism 9.0. Comparisons between two groups were made using two-tailed unpaired Student’s t tests; comparisons among multiple groups were made using one-way ANOVA followed by Tukey’s post hoc test. Significance was defined as * *p* < 0.05, ** *p* < 0.01, *** *p* < 0.001, **** *p* < 0.0001.

## 3. Results

### 3.1. HSPB1^S135F^ Impairs Neuronal Mechanoadaptation to Cyclic Stretch in a Stiffness-Dependent Manner

**Figure 1:**
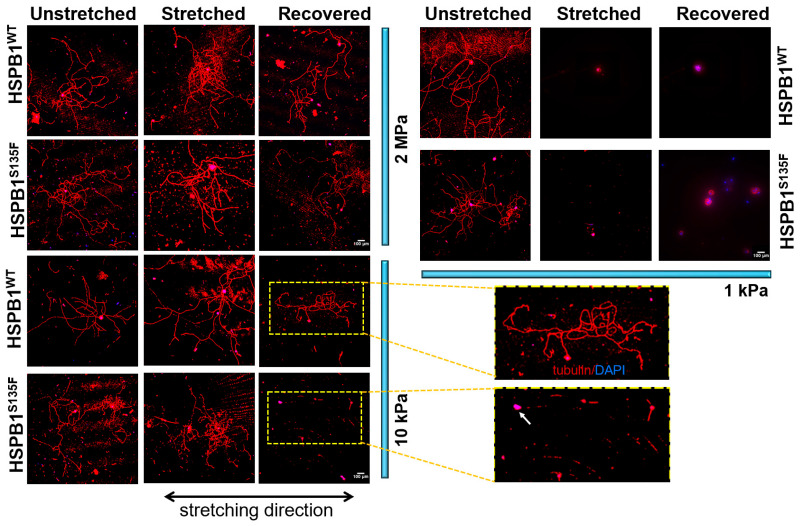
HSPB1^S135F^ impairs neuronal mechanoadaptation to cyclic stretch in a stiffness-dependent manner. Primary DRG neurons isolated from HSPB1^WT^ and HSPB1^S135F^ transgenic mice were cultured on PDMS substrates of defined stiffness and subjected to uniaxial cyclic stretch (10% strain, 0.1 Hz, 30 min, stretching direction: ↔). Cytoskeletal architecture was visualized by phalloidin staining (red). On rigid substrates (2 MPa), HSPB1^WT^ neurons displayed perpendicular reorientation and complete morphological recovery after 24 h, whereas HSPB1^S135F^ neurons showed persistent disorganization. On 10 kPa substrates, HSPB1^WT^ neurons retained adaptive capacity; HSPB1^S135F^ neurons underwent axonal fragmentation and shedding (soma without axons indicated by white arrow). On 1 kPa substrates, both genotypes exhibited irreversible structural disruption, with accelerated degeneration in mutants. Scale bar, 100 μm. Three independent experiments were performed, and representative images are shown.

Mechanical stress triggers adaptive cytoskeletal remodeling that is essential for maintaining neuronal integrity [31,32]. Previous studies have established that under high-frequency and high-amplitude stretch, actin polymerization drives the formation of stress fibers perpendicular to the stretch direction [33]. This reorganization facilitated axonal elongation along the perpendicular axis and is essential for maintaining mechanical homeostasis, thereby preserving cytoskeletal network integrity when neurons experience mechanical stress [34]. To determine whether the CMT neuropathy-associated HSPB1^S135F^ mutation compromises this adaptive response, we subjected primary DRG neurons from HSPB1^WT^ and HSPB1^S135F^ transgenic mice to uniaxial cyclic stretch on PDMS substrates of defined stiffness (1 kPa, 10 kPa, 2 MPa; Figure 1).

On rigid substrates (2 MPa), HSPB1^WT^ neurons displayed characteristic mechanoadaptive behaviors: transient actin retraction parallel to the stretch axis during active stretching, followed by perpendicular reorientation and complete cytoskeletal recovery after 24 h of rest (Figure 1). In contrast, HSPB1^S135F^ neurons on rigid substrates failed to reorient perpendicularly and exhibited persistent cytoskeletal disorganization even after recovery. On intermediate-stiffness substrates (10 kPa), approximating the mechanical microenvironment of muscle tissue, HSPB1^WT^ neurons maintained their adaptive capacity, whereas HSPB1^S135F^ neurons showed severe axonal fragmentation and complete loss of viable neurons after recovery. On soft substrates (1 kPa), mimicking CNS-like stiffness, both genotypes exhibited irreversible cytoskeletal disruption. Notably, the most pronounced genotype-specific difference was observed on 10 kPa substrates, suggesting that the pathological consequences of HSPB1^S135F^ are unmasked within stiffness ranges physiologically relevant to the peripheral nervous system.

These phenotypic observations were recapitulated in SH-SY5Y cells expressing HSPB1^WT^ or HSPB1^S135F^. On rigid substrates, HSPB1^WT^ cells exhibited transient contraction, perpendicular reorientation evidenced by a marked increase in the proportion of cells aligned at 60–90° relative to the stretch direction, as well as full area recovery during the recovery period; in contrast, HSPB1^S135F^ cells exhibited a delayed reorientation response (Supplemental Figure S1A,B). On 10 kPa substrates, HSPB1^WT^ cells successfully reoriented perpendicularly, whereas HSPB1^S135F^ cells displayed mechanical rigidity and impaired reorientation (Supplemental Figure S1A,B). On 1 kPa substrates, both genotypes lost mechanoresponsiveness and exhibited progressive atrophy (Supplemental Figure S1A–C). Collectively, these data demonstrate that HSPB1^WT^ confers mechanical resilience to neurons, and that the S135F mutation abrogates this neuroprotective function in a stiffness-dependent manner.

### 3.2. HSPB1^S135F^ Disrupts Stiffness-Directed Neurite Outgrowth and Focal Adhesion Maturation

**Figure 2:**
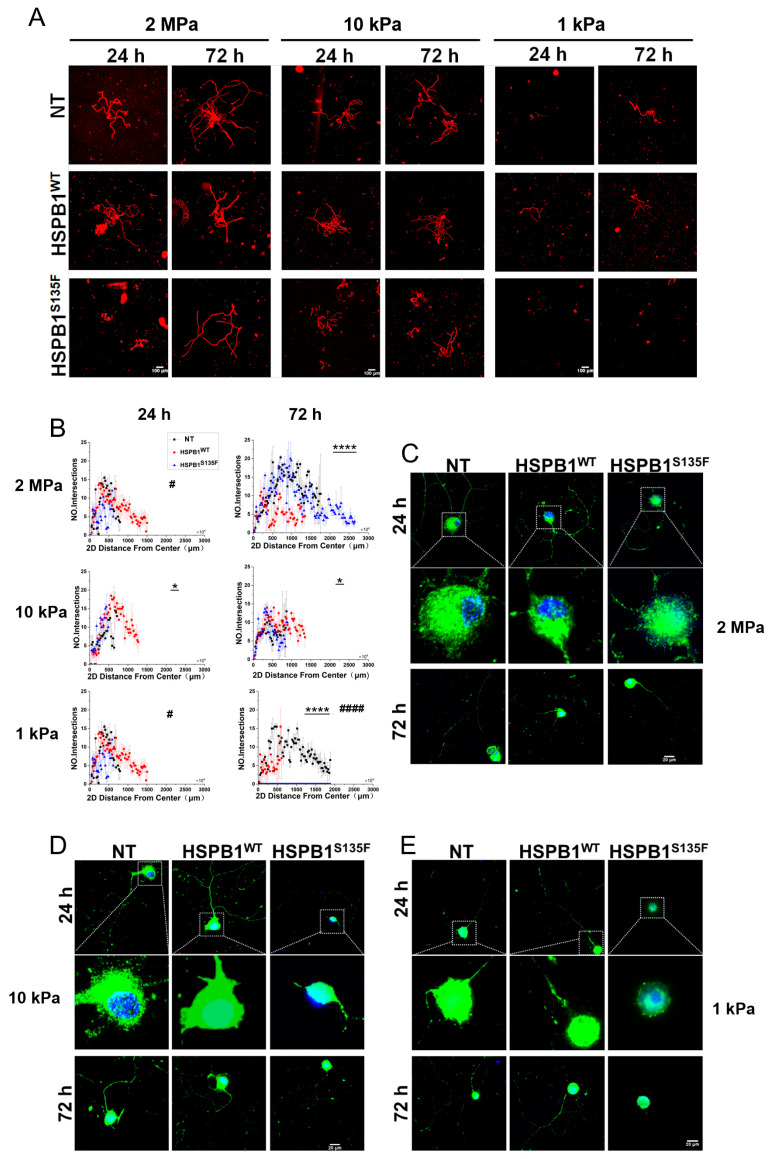
HSPB1^S135F^ disrupts stiffness-directed neurite outgrowth and focal adhesion maturation. DRG neurons from non-transgenic (NT), HSPB1^WT^ and HSPB1^S135F^ mice were cultured on PDMS substrates of indicated stiffness for 24 or 72 h. (**A**) Representative immunofluorescence staining for α-tubulin (red) reveals impaired neurite extension in HSPB1^S135F^ neurons. Scale bar, 100 μm. (**B**) Quantification of total neurite length per neuron (n ≥ 3); HSPB1^S135F^ versus HSPB1^WT^: * *p* < 0.05, **** *p* < 0.0001; HSPB1^S135F^ versus vector: ^#^ *p* < 0.05, ^####^ *p* < 0.0001. (**C**–**E**) Vinculin immunostaining (green) at 24 h shows well-organized, punctate focal adhesions at neurite tips in HSPB1^WT^ neurons, but diffuse, disorganized distribution in HSPB1^S135F^ neurons. Scale bar, 20 μm. Three independent experiments were performed, and representative images are shown.

Neuronal regeneration after injury requires precise sensing of ECM mechanical cues [35]. To assess whether HSPB1^S135F^ impairs stiffness-directed axonal growth, we cultured DRG neurons on tunable PDMS substrates and analyzed neurite outgrowth dynamics (Figure 2).

During the first 24 h of plating, HSPB1^WT^ neurons exhibited robust axon extension on all PDMS substrates (Figure 2A,B). Vinculin immunostaining revealed well-organized, punctate focal adhesions localized at axons and neurite tips across all stiffness conditions, providing a structural basis for efficient directional growth (Figure 2C–E). In striking contrast, HSPB1^S135F^ neurons displayed disorganized, diffuse vinculin distribution and severely impaired neurite initiation, particularly on soft and intermediate-stiffness substrates, (Figure 2D,E). Notably, the axon length of HSPB1^WT^ neurons showed no significant increase after prolonged culture (72 h) (Figure 2A,B), implying an early-stage role of HSPB1 in microenvironmental sensing and rapid growth initiation. Conversely, HSPB1^S135F^ neurons showed delayed but measurable elongation on rigid substrates (2 MPa, 10 kPa) by 72 h, yet completely failed to extend neurites on soft substrates (1 kPa), where HSPB1^WT^ and non-transgenic neurons retained partial growth capacity within the culture period (Figure 2A–B,E). These observations were corroborated in SH-SY5Y cells, with HSPB1^S135F^-expressing cells recapitulating the stiffness-dependent adhesion and growth defects (Supplementary Figure S2).

Collectively, these findings demonstrate that HSPB1^WT^ enables rapid, stiffness-responsive neurite outgrowth through proper focal adhesion assembly, whereas the S135F mutation disrupts this mechanotransduction pathway, leading to adhesion defects and stiffness-dependent regenerative failure.

### 3.3. HSPB1^S135F^ Abrogates Neuronal Differentiation and Maturation

Previous studies have demonstrated the critical role of HSPB1 in promoting SH-SY5Y cell differentiation into neurons [36]. To investigate the pathological consequences the HSPB1^S135F^ mutation on neurogenesis, we subjected SH-SY5Y cells expressing HSPB1^WT^ or HSPB1^S135F^ to a standardized RA-induced differentiation protocol on tunable PDMS substrates.

**Figure 3:**
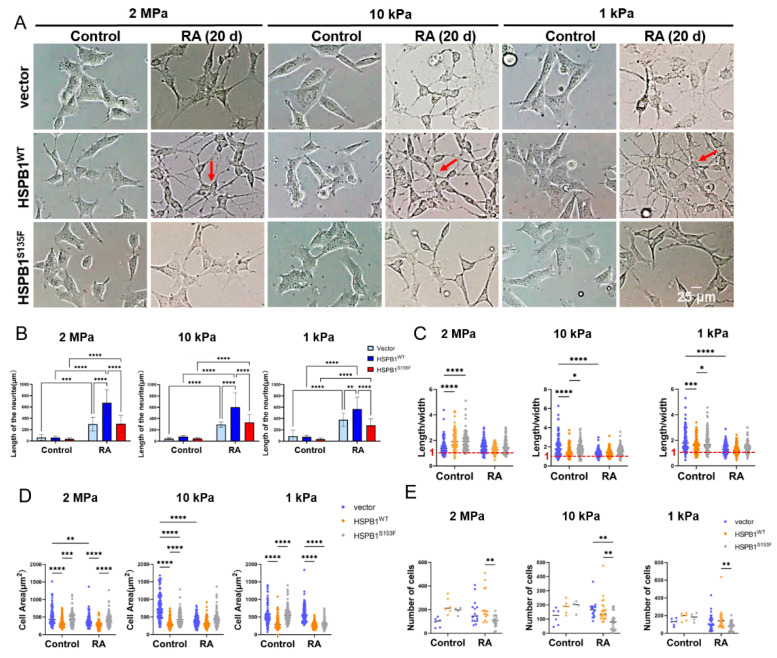
HSPB1^S135F^ abrogates neuronal differentiation and maturation. SH-SY5Y cells stably expressing empty vector (vector), HSPB1^WT^ or HSPB1^S135F^ were differentiated with retinoic acid (RA) on tunable PDMS substrates for up to 20 days. (**A**) Representative phase-contrast images before (Control) and after 20 days of RA induction. HSPB1^WT^ cells acquire neuronal morphology with extended neurites (indicated by red arrows); whereas HSPB1^S135F^ cells retain fibroblast-like morphology. Scale bar, 25 μm. (**B**) Quantification of neurite length per cell (*n* ≥ 12 cells per group). HSPB1^WT^ cells displayed significantly longer neurites than HSPB1^S135F^ cells across all stiffness conditions. Data are presented as mean ± SD from three independent experiments (biological replicates). (**C**) Circularity index calculated by the length-to-width ratio of somata, where values closer to 1 indicate a more rounded cell morphology. HSPB1^WT^ cells exhibited significantly higher circularity (i.e., more rounded somata) than HSPB1S135F cells (*n* = 100 cells per group). (**D**) Quantification of cell area (*n* = 100 cells per group). HSPB1^WT^ cells exhibited significantly reduced cell area compared with HSPB1^S135F^ cells. (**E**) Quantification of cell number per field. At least four randomly selected fields were analyzed per group. **** *p* < 0.0001, *** *p* < 0.001, ** *p* < 0.01, * *p* < 0.05.

After 20 days of RA induction, HSPB1^WT^-expressing cells exhibited robust differentiation capacity across all stiffness conditions, developing characteristic neuronal morphology including spherical somata, extensive neurite extension, and complex intercellular networks (Figure 3A–C). In contrast, HSPB1^S135F^ cells failed to acquire neuronal morphology, retaining flattened, fibroblast-like characteristics regardless of substrate stiffness. Quantitative morphometric analysis revealed that HSPB1^WT^ cells displayed significantly smaller cell areas and higher circularity indices, even prior to RA treatment, suggesting the priming role of HSPB1^WT^ in neurogenic commitment (Figure 3C,D). Notably, HSPB1^S135F^ cells showed markedly reduction in cell viability after RA treatment, indicating mutation-induced cytotoxicity during cell differentiation (Figure 3E).

**Figure 4:**
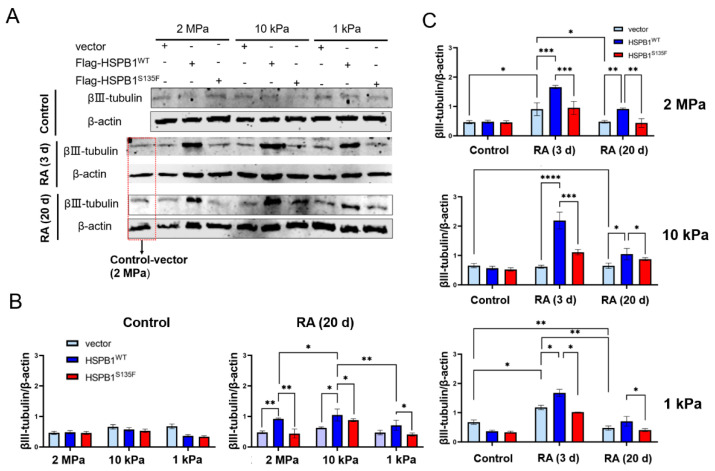
HSPB1^S135F^ blocks stiffness-optimized neuronal maturation. SH-SY5Y cells expressing empty vector (vector), HSPB1^WT^ or HSPB1^S135F^ were differentiated for up to 20 days on tunable PDMS substrates. (**A**) Western blot analysis of βIII-tubulin expression at days 0 (Control), 3, and 20. Dashed lines indicate baseline expression in undifferentiated controls. (**B**) Quantification of βIII-tubulin expression in undifferentiated cells and after 20 days of differentiation across all stiffness conditions. HSPB1^WT^ cells showed significantly increased βIII-tubulin expression on 10 kPa substrates, whereas HSPB1^S135F^ cells exhibited lower expression levels overall and lacked stiffness-dependent regulation. (**C**) Quantification of βIII-tubulin levels normalized to β-actin. At both day 3 and day 20, HSPB1^WT^ cells expressed significantly higher levels of βIII-tubulin than HSPB1^S135F^ cells, indicating enhanced neuronal differentiation. Data are presented as mean ± SD from three independent experiments (biological replicates); **** *p* < 0.0001, *** *p* < 0.001, ** *p* < 0.01, * *p* < 0.05.

To assess neuronal maturation, we monitored the expression of neuron-specific marker βIII-tubulin dynamics over 20 days of differentiation [37]. HSPB1^WT^ cells displayed upregulation of βIII-tubulin throughout the differentiation time course, with peak expression on 10 kPa substrates after differentiation (RA, 20d) (Figure 4A,B). In contrast, HSPB1^S135F^ cells maintained basal βIII-tubulin levels under all conditions, indicating complete differentiation blockade (Figure 4C). These data establish that HSPB1^WT^ promotes neuronal differentiation in a stiffness-optimized manner, and that the S135F mutation abrogates this pro-neurogenic function. This deficit in neuronal connectivity and network formation provides mechanistic insights into impaired nerve regeneration following PNI.

### 3.4. HSPB1^S135F^ Impairs Filopodia Formation and Nascent Adhesion Assembly During Early Neuritogenesis

**Figure 5:**
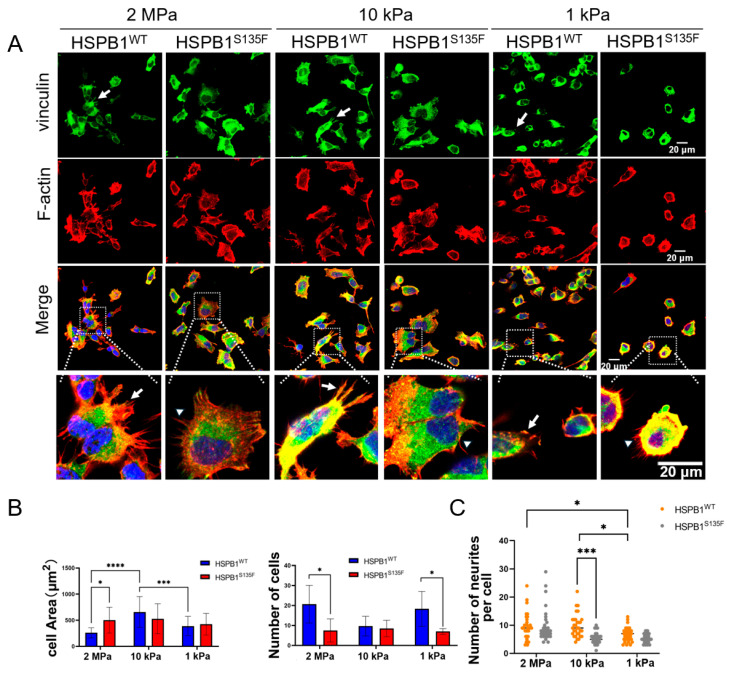
HSPB1^S135F^ impairs filopodia formation and nascent adhesion assembly during early neuritogenesis. SH-SY5Y cells expressing HSPB1^WT^ or HSPB1^S135F^ were differentiated with RA for 72 h on tunable PDMS substrates. (**A**) Representative confocal immunofluorescence images showing vinculin (green), F-actin (red), and nuclei (blue). White arrows indicate F-actin–rich filopodial protrusions with vinculin-positive tips in HSPB1^WT^ cells, indicative of nascent adhesion maturation. In contrast, HSPB1^S135F^cells exhibited rare filopodia formation (indicated by white triangles). Scale bar, 20 μm. (**B**) Quantification of cell area (*n* = 50 cells per group) and cell number per random field of view (three random fields per condition). Data are presented as mean ± SD. (**C**) Quantification of filopodia number per cell (*n* = 30 cells per group). On 10 kPa substrates, HSPB1^WT^ cells displayed significantly more filopodia per cell than HSPB1^S135F^ cells, indicating that the S135F mutation impairs stiffness-dependent filopodia formation. **** *p* < 0.0001, *** *p* < 0.001, * *p* < 0.05.

The failure of HSPB1^S135F^ cells to initiate neurite outgrowth prompted us to examine early cytoskeletal events during SH-SY5Y differentiation. Herein we further performed immunofluorescence analysis of F-actin organization and vinculin localization. After 72 h of RA induction, HSPB1^WT^ cells exhibited F-actin–rich filopodial protrusions at the cell periphery across all substrate stiffnesses, with vinculin colocalizing at the tips of these structures (Figure 5A, white arrows)—a hallmark of nascent adhesion maturation [38].

By contrast, HSPB1^S135F^ cells displayed a striking mechanosensitivity deficit: while they maintained filopodia formation on rigid substrates (2 MPa), filopodial structures were rare on soft substrates (10 kPa and 1 kPa), accompanied by cell body contraction and reduced cell number on 1 kPa substrate (Figure 5). These findings reveal that HSPB1^S135F^ disrupts the cytoskeletal-adhesion coupling required for filopodia stabilization, a defect that is unmasked specifically in compliant mechanical microenvironments.

### 3.5. HSPB1^S135F^ Causes Stage-Specific Dysregulation of TGase-Mediated Cytoskeletal Remodeling

Tissue transglutaminase (TGase) is a RA-responsive enzyme that stabilizes nascent neurites by crosslinking cytoskeletal proteins and modulating RhoA activity [39]. Given the profound neuritogenesis defects in HSPB1^S135F^ cells, we hypothesized that TGase dysregulation might underlie the observed phenotype.

**Figure 6:**
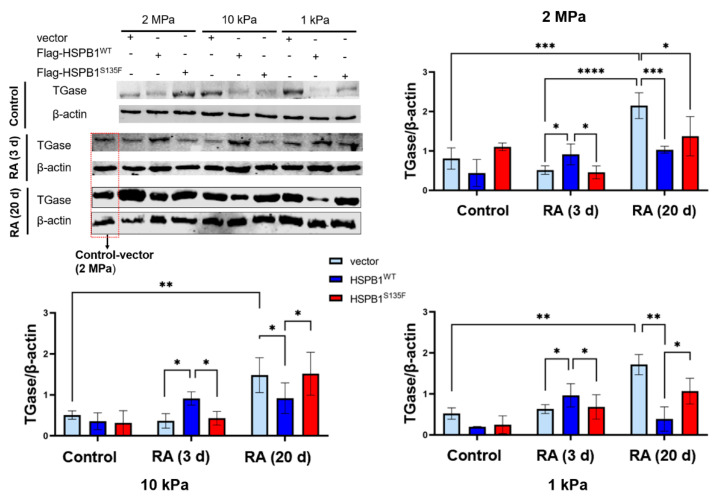
HSPB1^S135F^ causes stage-specific dysregulation of TGase. SH-SY5Y cells expressing HSPB1^WT^ or HSPB1^S135F^ were differentiated on tunable PDMS substrates; lysates were collected at days 0 (control), 3, and 20. Western blot analysis of TGase expression was performed. Quantification of TGase levels normalized to β-actin. HSPB1^WT^ cells exhibit robust TGase upregulation at day 3. HSPB1^S135F^ cells show attenuated early induction and paradoxical late-stage hyperactivation. Data are mean ± SD from three independent experiments (biological replicates); **** *p* < 0.0001, *** *p* < 0.001, ** *p* < 0.01, * *p* < 0.05.

During the initial differentiation stage (day 3) (Figure 6), HSPB1^WT^ cells showed robust upregulation of TGase across all stiffness conditions, consistent with its established role in stabilizing actin architectures and preventing premature neurite retraction (Figure 5A) [39]. in contrast, HSPB1^S135F^ cells displayed significantly attenuated TGase induction (Figure 6), correlating with their failure to form stable filopodia (Figure 5A). Remarkably, after prolonged differentiation (day 20), HSPB1^WT^ cells exhibited substantial downregulation of TGase compared with vector and HSPB1^S135F^ cells, indicating a stage-specific switch that facilitates growth cone motility and axon elongation. However, paradoxical TGase hyperactivation in HSPB1^S135F^ cells, suggesting that aberrant persistence of cytoskeletal stabilization physically constrains neurite extension at late stages (Figure 6). Of note, HSPB1^S135F^ cells showed TGase expression levels comparable to those of the vector control at both time points, suggesting that the S135F mutation abrogates the stage-specific regulatory function of HSPB1 on TGase, thereby phenocopying the empty-vector baseline.

These data reveal that HSPB1^WT^ orchestrates neuronal differentiation through precise, stage-specific modulation of TGase activity, and that the S135F mutation disrupts this temporal regulation—causing insufficient TGase during neurite initiation and failure to suppress TGase during elongation—thereby crippling regenerative capacity.

## 4. Discussion

The peripheral nervous system exists in a state of continuous mechanical challenge [40]. Peripheral axons are subjected to repetitive stretch from limb movement, must navigate ECM stiffness gradients during regeneration, and rely on precise cytoskeletal dynamics to maintain structural integrity [15,41]. Here we demonstrate that the CMT-causing HSPB1^S135F^ mutation fundamentally compromises the neuron’s ability to perceive and adapt to these mechanical cues, identifying defective mechanoadaptation as a potential pathomechanism in inherited peripheral neuropathy.

**HSPB1 is a biomechanical sensor that confers mechanical resilience.** Hoffman and colleagues, using mouse fibroblasts, established that HSPB1 is rapidly phosphorylated via the p38-MAPK2 pathway in response to uniaxial cyclic stretch, translocates to actin comet tails at sites of tension, and is required for stress fiber reinforcement and cell migration [6,7]. Our findings extend this paradigm to the nervous system: HSPB1^WT^ enables DRG neurons to reorient perpendicular to stretch direction, maintain cytoskeletal integrity after mechanical challenge, and execute stiffness-directed neurite outgrowth. Conversely, HSPB1^S135F^ neurons exhibit profound mechanoadaptation failure, manifesting as axonal fragmentation, adhesion disorganization, and regeneration arrest. Notably, these deficits are not cell-autonomous but are contextually unmasked on compliant substrates (1–10 kPa) that recapitulate the physiological stiffness of nerve microenvironments. This stiffness-dependent phenotypic penetrance offers a compelling explanation for the PNS-selective vulnerability in CMT: although HSPB1 is ubiquitously expressed, its pathogenic mutation only compromises function when cells are mechanically challenged within a specific stiffness range.

**Stiffness-optimized neurogenesis reveals a new dimension of HSPB1 function.** A central finding of this study is that HSPB1^WT^ promotes neuronal differentiation in a stiffness-dependent manner, with maximal βIII-tubulin expression and neurite complexity achieved on 10 kPa substrates that mimic muscle tissue stiffness. This observation aligns with the established principle that substrate stiffness directs stem cell lineage specification [18], but identifies HSPB1 as a previously unrecognized mediator of this mechanosensitive differentiation program. The complete differentiation blockade in HSPB1^S135F^ cells—even on optimal 10 kPa substrates—suggests that the mutation disables the cell’s ability to interpret ECM stiffness as a neurogenic cue. These findings have direct implications for neural tissue engineering: scaffolds designed for peripheral nerve repair must not only match the bulk stiffness of nerve tissue but also engage HSPB1-dependent mechanotransduction pathways to support neuronal maturation.

**Stage-specific TGase dysregulation: a mechanistic link between HSPB1 mutation and cytoskeletal dysfunction.** How does a point mutation in a small heat shock protein produce such profound and stiffness-dependent cytoskeletal defects? Although cyclic stretch was previously shown to induce HSPB1 phosphorylation [6], applying 5% stretch (0.2 Hz, 30 min) to SH-SY5Y cells—sufficient to drive cell reorientation (Figure S1)—did not alter HSPB1 phosphorylation, and the S135F mutation did not affect its phospho-status (Supplementary Figure S3). The absence of stretch-induced phosphorylation in our system likely reflects the lower mechanical stimulus used compared with earlier work (15%, 0.5 Hz, up to 1 h) [6], ruling out altered HSPB1 phosphorylation as a mechanism underlying the observed mechanoadaptation defects. Indeed, biochemical studies have shown that the S135F mutant, like wild-type HSPB1, undergoes phosphorylation by MAPKAP kinase 2 [42]. Thus, the S135F mutation does not impair the phosphorylation capacity of HSPB1, further supporting the conclusion that the cytoskeletal defects arise from other molecular mechanisms.

By tracking axon initiation and extension during SH-SY5Y differentiation, our data implicate the RA-responsive enzyme TGase as a critical downstream effector. Previous studies have shown that RA-induced differentiation of SH-SY5Y cells leads to upregulation of TGase expression [43,44]. Mechanistically, TGase catalyzes RhoA transamidation, activating RhoA-ROCK2 signaling to promote actin stress fiber assembly and focal adhesion maturation—processes that stabilize nascent neurites but must be downregulated to permit growth cone motility and axon elongation [39,45]. In this study, we show that HSPB1^WT^ orchestrates this temporal switch: TGase is robustly induced during early differentiation to stabilize filopodia (Figure 6, 3d), then suppressed at late stages to enable elongation (Figure 6, 20d). The S135F mutation abrogates this regulatory function, as HSPB1^S135F^ disrupts both phases—insufficient early TGase impairs filopodia stabilization, while aberrant late-stage TGase persistence constrains axon extension. This biphasic dysregulation represents a novel pathogenic mechanism in CMT and suggests that modulators of TGase pathway may offer therapeutic potential. For example, in the context of axonal injury repair, transient inhibition of TGase during the regenerative phase may enhance cytoskeletal dynamics and promote axon extension, offering a potential strategy to facilitate nerve regeneration for CMT patients.

**Limitations and future directions.** Several important questions remain. First, the molecular mechanism by which HSPB1^S135F^ dysregulates TGase expression is unknown. Our preliminary data suggest that HSPB1^S135F^ may aberrantly interact with TGase on soft substrate (1 kPa) (Supplemental Figure S4), but whether this reflects a direct chaperone-client relationship or indirect transcriptional dysregulation requires systematic investigation. Second, while our in vitro system recapitulates key stiffness parameters of the PNS microenvironment, peripheral nerves in vivo experience additional complexities—dynamic strain, inflammatory signals, and heterogeneous ECM composition [3]. Moreover, our PDMS-based culture system does not fully capture the three-dimensional topography, cell–cell interactions, or the dynamic mechanical cues (e.g., cyclic loading from joint movement) that peripheral axons encounter in native tissues, and these differences may either amplify or mitigate the mutant phenotype in vivo. Validation in ex vivo nerve explant models and in vivo nerve crush assays will be essential to establish the translational relevance of our findings. Third, our observation that HSPB1^WT^ optimally promotes neurogenesis on 10 kPa substrates raises the intriguing possibility that biomaterial stiffness could be therapeutically tuned to enhance nerve regeneration. Combining stiffness-optimized scaffolds with pharmacological activation of TGase-related pathways may represent a synergistic strategy for peripheral nerve repair.

## 5. Conclusions

This study establishes defective mechanoadaptation as a critical pathomechanism in HSPB1-linked peripheral neuropathy. We demonstrate that HSPB1^WT^ confers mechanical resilience and stiffness-directed regenerative capacity, while the S135F mutation disrupts this function through stage-specific dysregulation of TGase-mediated cytoskeletal remodeling. Given that CMT ultimately leads to muscle atrophy, which is tightly linked to loss of neuronal innervation, our findings suggest that the impaired cytoskeletal dynamics caused by HSPB1^S135F^ may contribute to muscle dysfunction indirectly through defective axonal maintenance. However, whether TGase-mediated cytoskeletal abnormalities also occur directly in muscle cells remains to be determined. Additionally, whether other disease-associated mutations in HSPB1 or in other CMT-causing genes act through similar TGase-dependent mechanisms is currently unknown, and our conclusions are therefore limited to the S135F variant studied here. Nevertheless, this study provides a conceptual and experimental framework for future investigations across the allelic heterogeneity of the disorder. Our findings reframe CMT pathogenesis from a purely cell-autonomous disorder to one in which genetic susceptibility and mechanical microenvironment interact to drive tissue-selective degeneration. This integrated biomechanical-molecular perspective not only provides new insights into peripheral nerve biology but also opens conceptual and therapeutic avenues for stiffness-targeted interventions in neurodegenerative disease.

## Data Availability

The original contributions presented in this study are included in the article/Supplementary Materials. Further inquiries can be directed to the corresponding authors.

## Supplementary Materials

The following supporting information can be downloaded at: https://www.mdpi.com/article/10.3390/cells15131216/s1, Figure S1. HSPB1^S135F^ impairs mechanoadaptation of SH-SY5Y cells to cyclic stretch in a stiffness-dependent manner. Figure S2. HSPB1^S135F^ disrupts stiffness-directed cell spreading and focal adhesion maturation. Figure S3. The HSPB1^S135F^ mutation does not alter HSPB1 phosphorylation, indicating alternative mechanisms for cytoskeletal dysregulation. Figure S4. HSPB1^S135F^ exhibits aberrant interaction with TGase during early RA-induced differentiation.
