# Supplementary material for: Neuropathy-Associated HSPB1 Mutant Impairs Neuronal Mechanoadaptation and Axonal Regeneration
Source: Cells. 2026 Jul 3;15(13):1216. doi: 10.3390/cells15131216 (PMC13359669; doi:10.3390/cells15131216)
Supplement: Supplementary file 1 [file cells-15-01216-s001.zip › cells-4388463-supplementary.pdf]

Supplementary material

Neuropathy-associated HSPB1 Mutant Impairs Neuronal Mechanoadaptation and Axonal Regeneration

Running title: HSPB1^{S135F} disrupts neuronal mechanoadaptation

Jiming Xie [1, 2, #], Ronglin Han [2, 3, #], Haidong Xu [1, 4], Zhiyu Li [2], Jingyi Zhao [1, 4], Ying Wan [2], Xianchao Pan [1, *], Juan Xing [1, 2, 4, *]

[1] School of Basic Medical Science, Bengbu Medical University, Bengbu, 233030, China;

[2] School of Basic Medical Science, Southwest Medical University, Luzhou, 646000, China;

[3] Department of Pathology, West China Hospital, Sichuan University, Chengdu, 610041, China

[4] Key Laboratory of Basic and Clinical Cardiovascular Diseases, Bengbu Medical University, Bengbu 233030, China;

[#] Jiming Xie and Ronglin Han contributed equally to this work.

***Correspondence:** Juan Xing (xingjuan217@bbmu.edu.cn); Xianchan Pan (panxc@bbmu.edu.cn)

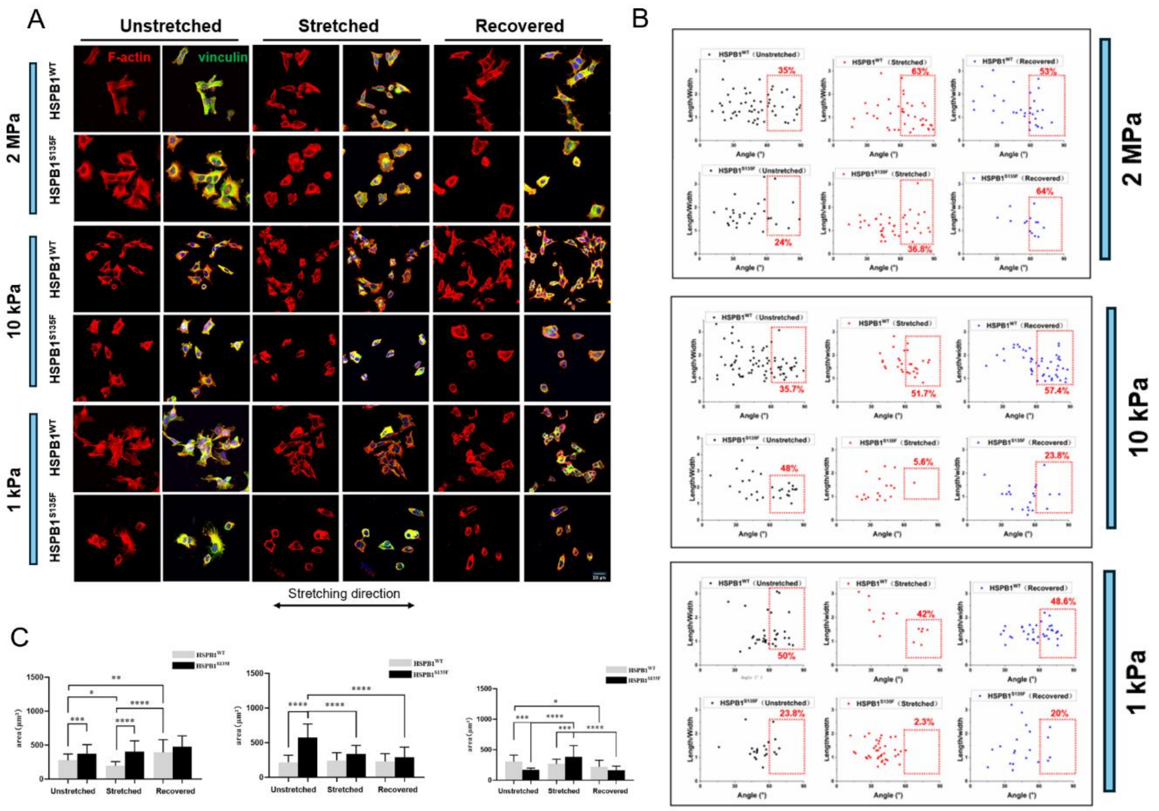

Figure S1. **HSPB1^{S135F} impairs mechanoadaptation of SH-SY5Y cells to cyclic stretch in a stiffness-dependent manner.** A. SH-SY5Y cells cultured on substrates of indicated stiffness were subjected to cyclic stretch, followed by immunostaining for vinculin (green) and F-actin (red). Scale bar, 20 μm. Three independent experiments were performed, and representative images are shown. B. Cytoskeletal morphology was analyzed using ImageJ. The aspect ratio (Length/width) and the angle between the cell long axis and the stretching direction were quantified. Regions enclosed by red dashed boxes denote cells with an orientation angle of 60–90° relative to the stretching direction, and the corresponding percentages are indicated in red font. C. Cell spreading area under each stiffness condition was measured using ImageJ ($n \geq 30$ cells per group). *, $p<0.05$; **, $p<0.01$; ***, $p<0.001$; ****, p<0.0001. HSPB1^{WT} cells exhibited a rapid response to cyclic stretch, characterized by perpendicular reorientation of the cell long axis relative to the stretch direction. On 2 MPa substrates, the proportion of cells oriented at 60-90° increased from 35% before stretch to 67% after stretch; on 10 kPa substrates, this proportion increased from 35.7% to 51.7%. In contrast, HSPB1^{S135F} cells showed stretching-induced orientation changes only on 2 MPa substrates, and this effect emerged only after the recovery period, with approximately 64% of cells exhibiting 60-90° orientation. Unlike HSPB1^{WT} cells, which underwent cytoskeletal rearrangement during stretching, HSPB1^{S135F} cells displayed delayed and stiffness-restricted mechanoadaptation.

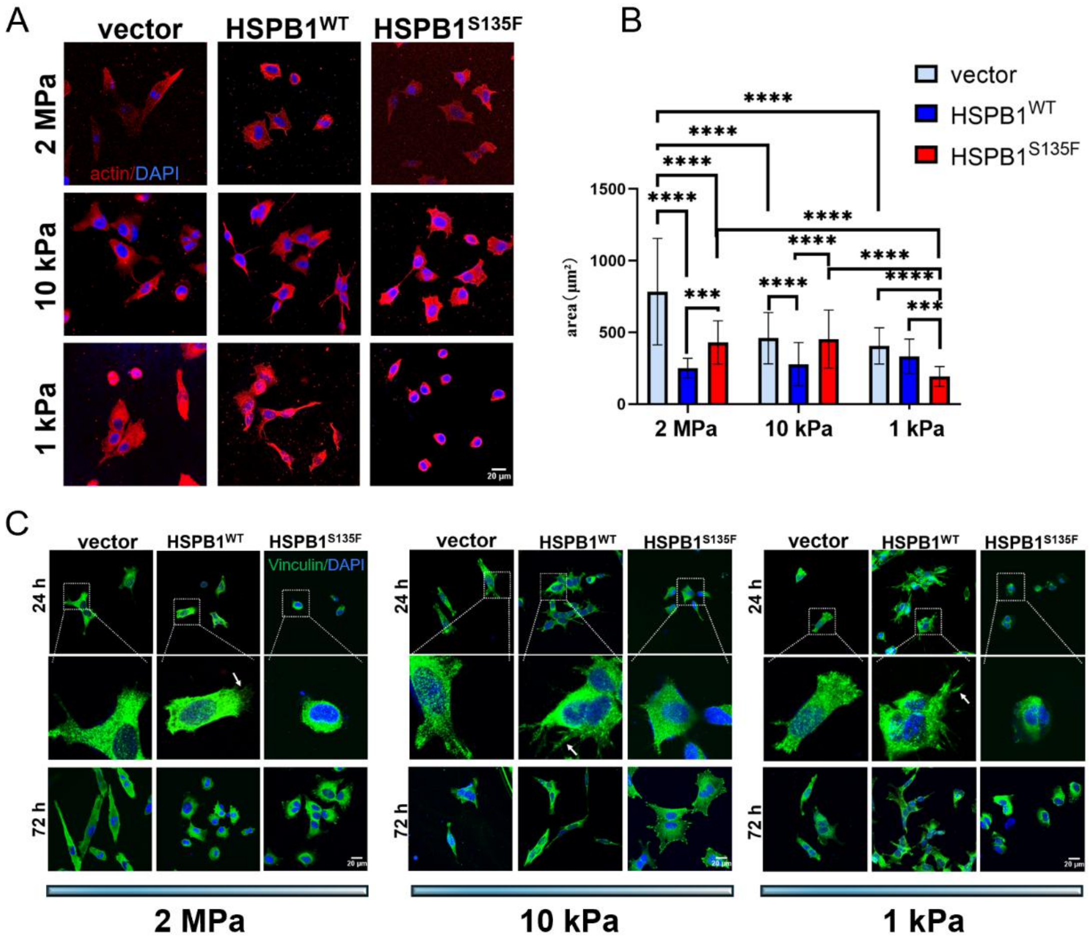

Figure S2. **HSPB1^{S135F} disrupts stiffness-directed cell spreading and focal adhesion maturation.**

A. SH-SY5Y cells cultured on substrates of indicated different stiffnesses were immunofluorescently stained for α-tubulin to assess the effect of matrix stiffness on cell morphology. Scale bar: 20 μm. Four independent experiments were performed, and representative images are shown. B. Quantification of cell spreading area reveled that HSPB1^{S135F} significantly impaired cell srpeading on soft substrates (1 kPa). Statistical analysis was performed using One-way ANOVA in GraphPad Prism 8. n>40 cells per group. *** $p<0.001$; **** $p<0.0001$. C. Vinculin immunofluorescence of SH-SY5Y cells cultured on substrates of varying stiffness at different time points, Magnified views of the perisomal region at 24 h are shown. HSPB1^{WT} enhanced perisomal focal adhesion assembly at early time points (24 h) (indicated by white arrows), whereas HSPB1^{S135F} exhibited reduced focal adhesion maturation. Scale bar, 20 μm. Four independent experiments were performed, and representative images are shown.

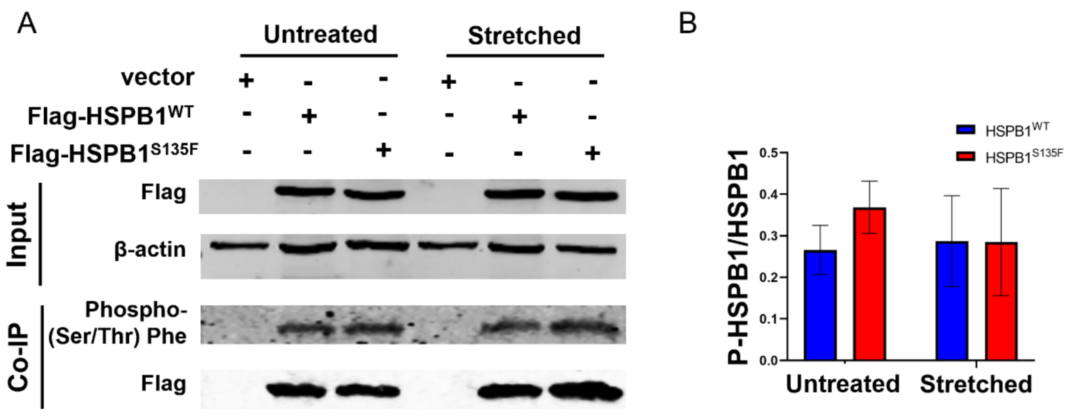

Figure S3. The HSPB1^{S135F} mutation does not alter HSPB1 phosphorylation, indicating alternative mechanisms for cytoskeletal dysregulation. **(A)** Western blot analysis of expression and phosphorylation of Flag-tagged HSPB1 in untreated or stretched cells subjected to cyclic stretch (5% strain, 0.2 Hz, 30 min). The expression of Flag-tagged HSPB1 was detected using anti-Flag antibody with actin as a loading control. The phosphorylation level of HSPB1 was assessed in co-immunoprecipitated (co-IP) samples prepared using Flag beads; equal amounts of immunoprecipitated proteins were analyzed for Flag (to confirm comparable pull-down) and for phospho-(Ser/Thr) levels. **(B)** Quantification of phospho-HSPB1 levels before and after cyclic stretch. No significant differences were observed between HSPB1^{WT} and HSPB1^{S135F}-expressing cells under either condition. Three independent experiments were performed.

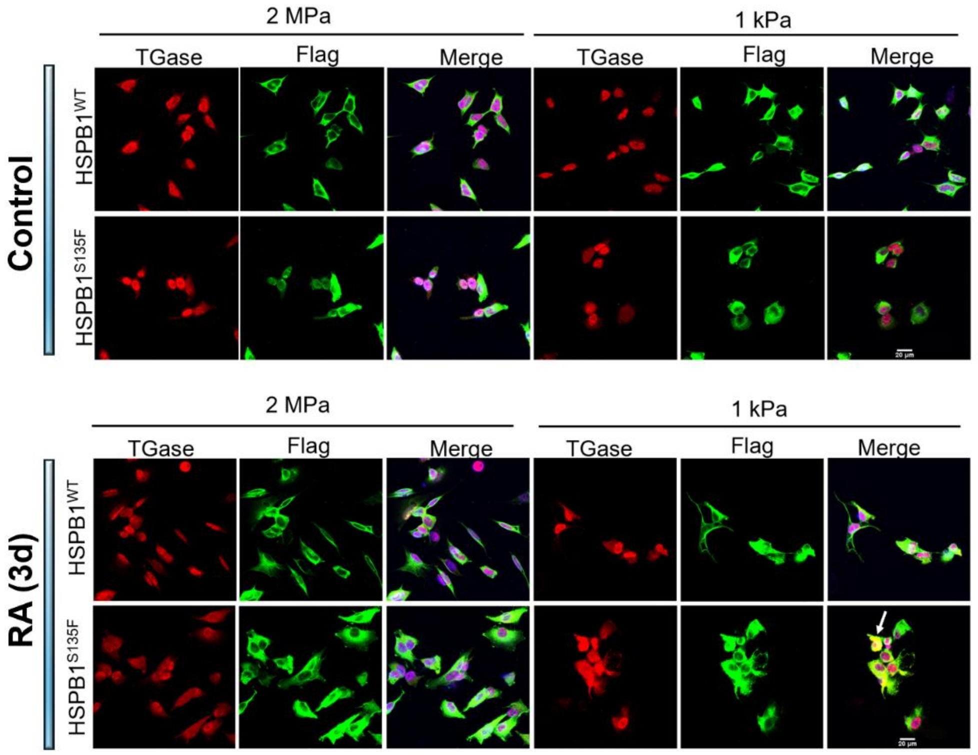

Figure S4. HSPB1^{S135F} exhibits aberrant interaction with TGase during early RA-induced differentiation. Immunofluorescence analysis of TGase distribution in SH-SY5Y cells cultured on

substrates of indicated stiffness (2 MPa, stiff; 1 kPa, soft) in control and RA-treated cells. Cells were immunostained for TGase (red) and Flag (green). White arrows indicate colocalization of HSPB1^{S135F} (Flag-tagged) with TGase, visualized as yellow signal. Three independent experiments were performed, and representative images are shown.
